# Public and Simulated Patient voice in the assessment of professionalism on healthcare degree programmes

**DOI:** 10.12688/mep.21119.1

**Published:** 2025-10-13

**Authors:** Jonathan D. T. Ward, Jose W. Jr. S. Cortezano, Jacob H. Chizzo, Katharine Heathcock, Katharine Weetman, Connie M. Wiskin

**Affiliations:** 1 Interactive Studies Unit, University of Birmingham College of Medicine and Health, Birmingham, B15 2TT, UK; 2 University of Birmingham School of Liberal Arts and Natural Sciences, Birmingham, B15 2TT, UK

**Keywords:** Medical Education, Professionalism, Assessment, Clinical Communication, Simulated Patients, Public perceptions, Undergraduate healthcare

## Abstract

**Background:**

In 2019, an International Working Group on the Assessment of Professionalism (IWGAP) identified a lack of patient voice in the literature relating to assessment of professionalism. This study sought to gather public perspectives on professionalism and associated methods for assessment.

**Methods:**

A mixed-method study was conducted to understand public perceptions of healthcare student professionalism and how these perceptions might inform future assessment practices. One hundred street surveys with members of the public were analysed and results were used as stimuli during two focus groups with simulated patients (SPs). Focus-group data were analysed in an iterative process to identify themes.

**Results:**

While 97% of survey respondents were in favour of assessing professionalism for undergraduate healthcare students, preferences for formal or informal assessments varied. ‘Benevolence’ and ‘competence’ were considered key components of professionalism, with ‘Showing respect for patients and colleagues’ ranked as the most important aspect. Seven focus-group themes were identified: presentation, attitudes and values, patient relationship and centredness, trustworthiness, timekeeping, knowledge, and formality and mode of assessment.

**Conclusions:**

While the public recognises the need to assess professionalism, healthcare programmes should re-assess the focus of examinations, which might prioritise aspects of professionalism deemed less important by patients.

## Practice points

- Public perception is overwhelmingly in favour of assessing professionalism, although preferences for formal or informal methods of assessment vary
- Perhaps contrary to current assessment foci, respect for patients and colleagues and other relationship-based domains were considered higher priorities for professionalism than aspects such as appearance, attendance and timekeeping
- A combination of assessment methods, including observation in the workplace, regular tutor monitoring and observation of mock consultations, was considered most appropriate for assessing professionalism
- Further research to explore patient and public perceptions is required, but should acknowledge the public’s potential lack of detailed understanding of assessment practices in medical education

## Introduction

Professionalism is widely accepted as core to healthcare education programmes and often features directly or indirectly in educational guidelines, competency frameworks and standards of national organisational regulatory bodies ^1– 4^. However, defining what precisely embodies professionalism has been a subject of discourse for several decades; consensus for how to universally define this complex construct remains elusive ^5, 6^. Nevertheless, there are commonalities underpinning the constitution of professionalism that appear in the literature: honesty, integrity, appropriateness, compassion and empathy, accountability, altruism, good communication and interpersonal relationships, organisation, and respect ^7– 11^.

The literature often frames professionalism as a competency—a set of skills that can be taught, applied, and objectively measured at the institutional level ^12– 14^. However, other approaches highlight its complexity ^6, 8^, complicating objective assessment. Goldie ^15^ views professionalism as a multi-dimensional construct encompassing individual, interpersonal, and institutional contexts, suggesting that assessments should address attitudes, values, and behaviours, not just ethical adherence. Epstein and Hundert ^16,p.226^ define professional competence as ‘The habitual and judicious use of communication, knowledge, skills, reasoning, emotions, values, and reflection in daily practice’. Similarly, the Health and Care Professions Council ^17^ describes professionalism as a meta-skill, requiring situational awareness and contextual judgment. While international reviews have explored assessing professional competencies ^6, 18– 20^, further investment is needed.

Over the past decade there has been movement towards Professional Identity Formation (PIF) ^21^, a broader belief system to reinforce individuals’ perceptions of themselves as professionals rather than reduce professionalism to a checklist of traits, often seen in behaviour-based competency models in healthcare curricula. Professional identity is a process that is fluid and ongoing ^15, 22^. It can be interpreted as a ‘representation of self, achieved in stages over time during which the characteristics, values, and norms of the medical profession are internalized’ ^23,p.1447^.

Although the importance of PIF is increasingly understood, integrating it into healthcare training programmes and curricula — then measuring it — remains a challenge. The teaching of professionalism is complex, requiring strategies that explicitly as well as implicitly develop a learner’s knowledge, attitudes, judgment and skills ^24^. Nonetheless, there seems to be broad consensus that professionalism should be assessed longitudinally with multiple instruments (otherwise referred to as multimethod or triangulation), both formative and summative, and several assessors with juxtaposition of feedback and/or reflection ^5– 7, 15, 25, 26^.

Many studies ^9, 13, 27– 29^ have examined what constitutes professionalism and how it develops, as perceived by students, patients, and educators. These insights have shaped healthcare education practices globally ^8, 18, 26, 30– 36^. In the USA, for example, Stern and Papadakis ^37^ categorize teaching professionalism into three areas: setting expectations, providing experiences, and evaluating outcomes. Evaluation includes both formative and summative assessments, such as multiple mini interviews, faculty assessments on placement, peer reviews, and patient perceptions of physician conduct. In Canada, Cruess and Cruess ^32^ discussed opportunities in teaching contexts for reflection and internalisation of professional values and behaviours. Others have focussed on evaluating the reliability and validity of the assessment tools themselves (e.g. ‘clinical conscientiousness index’, such as Finn *et al*. ^38^ and Kelly *et al*. ^39^). Notably, OSCEs (Objective Structured Clinical Examinations), observations, incident reports, case-based discussions and supervisor reports are widely used to assess professionalism ^5, 15, 20^. There is also a safety prerogative, given the critical relationship between professionalism, trust and clinical outcomes ^40, 41^.

The updated Ottawa Consensus Group Statement ^42^ refines previous recommendations on assessing professionalism ^43^. The authors express concern that, despite incorporating patient perspectives in areas like quality improvement and patient safety, a significant gap remains: integrating the patient voice in assessing professionalism and addressing conflicts between individual and institutional values.

This study addresses the gap by incorporating public perspectives to enhance discussions on assessing professionalism in undergraduate healthcare programmes. We hope that findings inform current practice and guide educators and assessment leads in re-examining curricula and assessments. The results offer recommendations for developing and evaluating educational activities related to professionalism. Furthermore, this project could inform larger-scale studies over multiple sites around and beyond the West Midlands, UK.

## Materials and methods

A two-part mixed-method study, comprising a street survey and follow-up focus groups.

**Part 1:** Data were gathered from a convenience sample of 100 members of the general population in July 2023. Location was an outdoor public right-of-way, at the intersection of a university, NHS hospital and train station. Passersby were invited to spend 5–10 minutes participating in an oral survey about healthcare student professionalism, using a semi-structured questionnaire ^44^ designed by the co-investigators. Questions were a mix of closed, list, ranking and free text. Data were anonymous, bar some broad (non-identifiable) demographic categorisation. Responses were recorded by the investigator directly into electronic tablet forms ^45^. There was no video/voice recording. Results here informed Part 2.

**Part 2:** The results from Part 1 ^46^ were presented to two focus groups of 6–7 professional Simulated Patients (SPs) to discuss how public feedback could inform the assessment of professionalism in academic curricula, evaluate current needs, and explore ways to improve assessments.

Duration was two hours, face-to-face, in a university tutorial room. Groups were audio recorded for thematic analysis, using the Clarke and Braun Model ^47^. The co-investigators work with 100 internally recruited/trained SPs, who — as suppliers — support Clinical Communication and Professionalism teaching and assessment on the university’s medical/healthcare degree programmes. Recruitment was by open expression of interest, then purposive from positive replies to manage demographic representation. Data were presented, followed by facilitated discussion from structured prompts.

### Ethical considerations

**Permissions:** The project was approved by the University of Birmingham Staff Projects Committee, University of Birmingham, with reference ERN_1175-May 2023.

**Safety:** The investigation included an undergraduate research student, funded internally through the University of Birmingham Liberal Arts and Natural Sciences scheme. The student was supported throughout, including close-by access to a staff co-investigator and secure building during public data collection.

**Informed consent:** For Part 1, participation in the survey indicated consent; clearly stated to participants. The clause was read aloud by the investigator at the outset, or read by the participant, as preferred. A participant information summary was offered in oral or written form and oral informed consent was obtained due to the nature of the survey (outdoor, in street interviews with passers-by). All participants were over 18 years of age. For Part 2, participation in the focus group indicated consent. This was stated firstly in the recruitment email, secondly to those who came forward, with a copy of the participant information and consent form ^48^ included, and thirdly at the focus group itself, prior to commencement. All participants provided written consent prior to attending the focus group sessions.

**Confidentiality:** No public demographic data other than age-bracket were recorded. In focus groups participants were obviously visibly identifiable but not known to the student interviewer. No names appear in the data. In transcription from audio recording any identifiable references were removed.

**Data storage**: In line with University of Birmingham governance, data are held on secure restricted-access approved devices, for ten years.

**Inclusion and exclusion criteria:** Participants for Parts 1 and 2 were adults over 18. Interested survey participants were excluded if they were currently a health professional/healthcare student to avoid bias from experience of existing assessment processes. The choice of oral or written questions addressed literacy and vision sensitivity.

**Withdrawal:** As Part 1 had no personal data recorded later withdrawal was impossible (no means of cross-referencing). Part 2 SPs could withdraw during the group. Transcripts were pseudo-anonymised, preventing retrospective withdrawal — other than a self-identified specific phrase.

**Sensitivity:** Questions related to ‘if and how students should be assessed on their professionalism’. There were no invasive/personal questions; distress risk was minimal.

**Reward:** Survey participants were offered a claim link to a £5 voucher as a ‘thank you’. This was after the survey, so not an incentive. Focus group participants’ travel was reimbursed. They were given a £50 voucher after the event to acknowledge expertise and sacrifice of paid work opportunity. This rate aligned with NIHR PPI guidelines.

## Results

### Public survey results – quantitative and free text

The 100-survey target was met. Participant age range was 18 to 75+, with 70% aged between 18 and 54 years.

***Public ‘top of mind’ responses to the word ‘professionalism’***

Respondents were invited to give up to three open responses to the question, *‘Thinking about healthcare professionals, what first comes to mind when I say the word ‘Professionalism’?’*. In theming, competence 18.2% (n=29) and benevolence 18.2% (n=29) scored highest. Good communication 7.5% (n=12), good attitude/manners 7.5% (n=12) and patient-centredness 5.7% (n=9) were also considered positively. Also considered relevant were aspects of status, relating to being a healthcare professional/in a healthcare institution at 6.9% (n=11), being educated 6.3% (n=10) and being experienced 5.7% (n=9).

Negative comparisons were made by 4.4% (n=7) of respondents, including lack of professionalism, lack of knowledge, overwork and underpayment for health care professionals amongst others ( Table 1 ^46^).

**Table 1 46 .** Q: Thinking about healthcare professionals, what first comes to mind when I say the word ‘Professionalism’?

Themes	Frequency (n)	Percentage (%)
**Competence** (knowledgeable, know what they’re doing, good at their job, top of the game, expert in the field, conscientious, smart, quality, good result, diligence, skills, efficient, expertise)	29	18.2
**Benevolence** (caring, compassionate, dignity, vocation, empathy, friendly, ethics, kind, integrity, nice, helpful, service, polite)	29	18.2
**Good communication** (willing to listen, receptiveness, meticulous at addressing needs, appropriate tone with patients, communicate science, informative, speak to you with respect, doesn’t jump to conclusion)	12	7.5
**Good attitude and manners** (attitude, manners, conduct, the way you conduct yourself, mannerism, boundaries, well-mannered, bed-side manner, code of conduct)	12	7.5
**Healthcare professional and institutions** (doctors, nurses, NHS, medical staff)	11	6.9
**Educated** (study, education, degree, training, qualified)	10	6.3
**Experienced** (experienced, specialised, in control, structured, leadership, responsibility)	9	5.7
**Patient-centredness** (patient care, confidentiality, trust, deal with patient, how they treat people, attitude to patient)	9	5.7
**Negative comparisons** (lack of professionalism, lack of knowledge, tiring, complicated, overworked, underpaid)	7	4.4
**Respect** (respect, not to condescend, respect privacy)	5	3.1
**All others combined** (under 3.5%)	26	16.4
**Total**	159 *	**99.9**

* Some respondents gave multiple answers

***Attitudes towards assessing students’ professionalism***

Almost all respondents (97%) considered that students should be assessed for their professionalism but were divided about whether that assessment should be a formal (25%) or informal (40%) assessment or a combination of both (31%).

***Importance of aspects or behaviours of professionalism to be assessed***

Respondents identified the five most important aspects of professionalism which could be assessed from a list of ten aspects. They were then asked to rank their choices from 1 to 5, 1 being the most important and 5 the least important. The most frequently chosen responses, with valid percentages for 497 responses in total, were for interpersonal aspects and behaviours which demonstrated respect for others (16.5%), maintaining relationships with patients (14.7%) and honesty (14.1%). However, personal aspects and behaviours, such as maintaining up-to-date knowledge (14.1%), good hygiene (13.1%) and good timekeeping (12.7%) were the next most frequently chosen ( Table 2 ^46^). 

**Table 2 46 .** Q: Thinking about healthcare students, which of these aspects or behaviours would you choose as the five most important aspects of professionalism to assess?

	Aspects/behaviours of professionalism	Frequency of overall responses (n)	Percentage (%)	Valid Percentage (%)
Valid	Showing respect for patients and colleagues	82	16.4	16.5
	Maintaining a good relationship with patients	73	14.6	14.7
	Demonstrating honesty (if an error is made or copying others' work)	70	14.0	14.1
	Keeping knowledge up to date	70	14.0	14.1
	Maintaining hygiene	65	13.0	13.1
	Timekeeping (arrival time at class/work)	63	12.6	12.7
	Being a good role model	29	5.8	5.8
	Attending class/work placements consistently	25	5.0	5.0
	Paying attention to appearance	18	3.6	3.6
	Using social media appropriately	2	0.4	0.4
	**Total**	**497**	**99.4**	**100.0**
Missing *	System	3	0.6	
	**Total**	**500**	**100**	

* Note that 3 responses are missing due to a system error

*Showing respect for patients and colleagues* was ranked first by 36% of respondents, double the next answer which was *Keeping knowledge up to date* at 18%. *Maintaining a good relationship with patients* was most frequently ranked second (28%), *Demonstrating honesty (if an error is made or copying others' work)* was most frequently ranked third (17%), *Timekeeping (arrival time at class/work)* was most frequently ranked fourth (21%) and *Maintaining hygiene* was most frequently ranked in fifth place (22%). While *Keeping knowledge up to date* does not appear most frequently in any ranking position, it is the only aspect which scored above 10% across all rankings ( Table 3 ^46^).

**Table 3 46 .** Frequency of rankings from 1 to 5 (where 1 is highest ranked and 5 is lowest ranked).

Aspects/behaviours of professionalism	Frequency of rank 1 (n/%)	Frequency of rank 2 (n/%)	Frequency of rank 3 (n/%)	Frequency of rank 4 (n/%)	Frequency of rank 5 (n/%)
Showing respect for patients and colleagues	**36/36**	21/21	14/14	8/8	3/3
Maintaining a good relationship with patients	13/13	**28/28**	14/14	8/8	10/10
Demonstrating honesty (if an error is made or copying others' work)	9/9	12/12	**17/17**	19/19	13/13
Keeping knowledge up to date	18/18	16/16	13/13	12/12	11/11
Maintaining hygiene	8/8	9/9	14/14	12/12	**22/22**
Timekeeping (arrival time at class/work)	13/13	9/9	12/12	**21/21**	8/8
Being a good role model	0/0	2/2	7/7	4/4	16/16
Attending class/work placements consistently	2/2	1/1	5/5	9/9	8/8
Paying attention to appearance	1/1	1/1	3/3	6/6	7/7
Using social media appropriately	0/0	1/1	0/0	0/0	1/1
Missing response	N/A	N/A	1/1	1/1	1/1
**Total**	**100**	**100**	**100**	**100**	**100**

* Note that 3 responses are missing due to a system error (1 answer each for ratings 3, 4 and 5)

***Potential actions in response to a finding of lack of professionalism***

Respondents were shown eight potential courses of action in response to the question, *‘Imagine a student has the correct knowledge to progress to the next stage of their course but has been found to lack professionalism, what do you think should happen to this student?’* and asked to choose up to three most suitable actions. Having to attend a review meeting with a teacher/tutor before continuing studies (42.6%) was most frequently chosen as the most suitable action, with all other actions scoring under 12%. No respondents chose the most extreme courses of action — immediate removal from the degree programme or no consequences if clinical knowledge was sufficient.

‘Other’ responses totalling 21.7% were single answers, including suggestions such as further training, correctional courses, extra tuition, more face-to-face learning with patients, one-to-one guidance, robust mentoring and professional development support. Specific suggestions included a one-day course with six-month review or a month-long course, and two respondents felt that professionalism would develop with further experience and maturity.

***Current assessment of professionalism***

Respondents were shown nine types of professionalism assessment methods and asked to rank the three most effective. Observation in the workplace (31.3%), regular meetings with teachers/tutors (19.3%) and observation of mock consultations with patients (18.0%) were the most frequently chosen. Team based exercises or activities (10.3%) and self-reflection (10.0%) were less frequently chosen. All other responses scored 6% or less.

### Focus group results – qualitative thematic analysis

13 simulated patients participated across two focus groups.

Data were initially coded by JWC, resulting in 23 codes: showing respect, hygiene, appearance and dress, infection control, timekeeping, honesty, role-model, twenty-first century healthcare, changing role of doctor, e-professionalism, state of UK healthcare, patient-centred care, respect for the role, expectations, confidence vs. arrogance, defining professionalism, learning through experience, impact of professionalism, assessing professionalism, assessor’s behaviour, student support for professionalism, formalised assessment, and improving assessment of professionalism. Independently, data were analysed and coded in NVivo by KW, generating 18 codes — with some overlap: appearance, attendance, attitudes and values, body-language, competency and knowledge, empathy, formality and mode of assessment, honesty, humanity, hygiene, non-judgement, patient relationship, patient safety, respect, role-modelling, self-reflection, timeliness, and trustworthiness. The consolidation was adjudicated and further reviewed by JW. Disagreements were discussed to form the final seven themes described below.

***Theme 1: Presentation***

Notable was the disproportionate amount of time spent discussing appearance and dress in the focus groups compared to attention received in the public surveys. This theme encompassed three areas — appearance, body language, and hygiene. Appearance tended to focus on dress and clothing, and its impact: *‘So if somebody isn’t dressed appropriately, then you don’t take the work seriously because that gives you some confidence on the way they present themselves.’* [P5], *‘there needs to be some sort of thought about what we wear and the impact that it can have”* [P4]. There was acknowledgement that what constitutes professional appearance in healthcare has evolved: *‘there is a changing in societal norms now, you know, healthcare professionals, having tattoos or piercings or things like that’* [P1]. However, there was acknowledgement that preference for expensive/designer attire is ‘elitist’. Most caveated with clarification that societal changes and expectations do not undermine the professionalism of the healthcare clinician: *‘I would feel as safe with a doctor with facial piercings or tattoos as I would who was dressed very smartly.’* [P1], *‘…it always strikes me it's… the range of different outfits that people would wear.’* [P2], *‘…it’s like how you were saying with tattoos and piercings. You’d still value that person as a professional, you know, because they’ve gone up that ladder… To do their job.’* [P6].

Body-language coding focussed on behaviours of students with neurodivergence, such as lack of eye contact: *‘What struck me is with neurodiversity… eye contact for them is not comfortable and it’s really difficult and they may be excellent in different ways but, quite tricky’* [P12], *‘I had to train [PERSON] to look at people’s shoulders, don’t be rude…’* [P11]. Hygiene comments tended towards infection-control, in light of COVID-19, as opposed to personal hygiene or self-care: *‘it's maintaining hygiene, it’s putting the patient in danger in hospital and hygiene should be paid attention to, get back to patient safety.’* [P8], *‘in terms of best practice, in terms of how you wash and prepare yourself to to to [sic] consult with the patients as opposed to your personal hygiene’* [P9].

***Theme 2: Attitudes and values***

This theme, encompassing colleagues and service users, was prevalent throughout both groups. Some participants acknowledged unconscious bias and the effect of a student’s background on their professionalism: *‘…is it coming from that their family background, that’s how it was in the family home?’* [P8]. There was distinction and discussion of how attitudes can impact inter-professional relationships as well as professional-patient ones: e.g., *‘one of the students said “well she’s just a nurse… we've got authority at the end of the day”’* [P11]. The difficulty of assessing attitudes and values was raised, including the challenge of distinguishing between positive and negative attributes such as ‘confidence’ and ‘arrogance’.

One positive attribute of particular interest was ‘respect’, *‘showing respect to your colleagues as well as your patients, you know, at all times’* [P6], which sometimes overlapped with being ‘non-judgemental’ or free from prejudice regardless of *‘who they are, their age, where they’ve come from’* [P1]. Lack of respect was conceptualised as being caught using a mobile phone, not treating patients well or equally, not listening and impoliteness, with questions raised as to whether *‘black humour*’ is professional or not:

*I think it's unrealistic to imagine that they’re not gonna talk about things they've experienced and in terms of letting off steam… in a safe environment where people understand. It isn't malicious… It's just about the situation they’ve been through, them needing to express that.* [P4]

***Theme 3: Patient relationship and centredness***

The patient relationship, and its importance, was discussed considering the public ranking this second in the survey results. SPs remarked on the correlation between patients not feeling listened to and complaints in the NHS: *‘you know that, that is what people are most troubled by when they have a bad experience’* [P9]. Two factors described as integral to the therapeutic and clinical relationship were simply ‘ *humanity and kindness’* [P8] as well as ‘empathy’ by others, *‘how do you become a kind of neutral but empathetic and approachable human being’* [P1].

***Theme 4: Trustworthiness***

The importance of trustworthiness and honesty were evenly discussed in both groups. This covered attributes such as being truthful and trustworthy, feeling safe, openness, integrity, admitting mistakes, not plagiarising, ethical practice, and generally, *‘doing the right thing’* [P11] e.g. *‘whistleblowing’*. SPs also discussed environments, such as teaching and learning settings, which are conducive to honesty and disclosure: *‘…it’s like a safe zone where people can be honest. It’s like a healthy discussion, almost more like a moral debate, isn’t it? And “People can see why I find this difficult”, “That really upsets me”, or “Why I get really upset” and you get the very good facilitator, the GP’* [P8].

***Theme 5: Timekeeping***

Attendance and timeliness were briefly discussed in both groups, including the importance of attending placement: *‘if you don’t turn up for your work placement, potentially you’re letting the team down’* [P3]. Although the importance of timeliness was noted, so was appreciation that this may be *‘out of healthcare professionals’ hands most of the time’* [P1] and a result instead of staffing shortages and system design.

***Theme 6: Knowledge***

SPs referred to the importance of knowledge and competence as integral to professionalism and inseparable from patient safety: *‘you've got to have that, that professionalism and kind of have those extra skills you know, to deal with that. So, it does need to be worked, worked upon in a kind humane way…, want to build their expertise’* [P7]. It was also remarked that risks can be encountered actively or passively: *‘And is it a deliberate action, or an accidental… action in ignorance or inaction for that matter, or not aware they should be doing this’* [P9]. However, there was not universal agreement on this area with some describing a separate entity, *‘But we're talking about professionalism, not medical knowledge’* [P10].

***Theme 7: Formality and mode of assessment***

Given that this was prompted it was an anticipated theme: *‘I would love to see a more formal element on professionalism because I think it really helps to drive that sense of how important it is’* [P4]. Types of assessment for professionalism mentioned included observation in workplace and *‘shadowing somebody’*, mock consultations, meetings with tutors, self-reflection, peer assessment, role player assessment, and exams or OSCEs. Generally, multi-method assessment or a hybrid approach were rated positively: *‘You get lots of good things from, from each aspect’* [P8]. Role modelling was discussed as both an attribute of professionalism itself: *‘…things like being a good role model, I think that goes back to a kind of modern idea of a doctor and goes into the idea of how they live their lives personally’* [P4]; a teaching and peer support method: *‘if fifth years just having conversations with first years, it would give them a wealth of knowledge’* [P1]; and an assessment method and measure: *‘role modelling within the role play situations’* [P4]. Self-reflection was repeatedly raised: *‘you need to have the self-awareness of what you're doing and your impact’* [P1]. This was highlighted in contexts where the student can learn through mistakes and errors: ‘ *We're humans ourselves, we make mistakes… as long as done in good faith’* [P11] and *‘The challenge with professionalism is often it's uncomfortable things, but we don't know when to talk about it.’* [P10]

Assessment formality was raised; both groups challenged the three respondents who answered ‘no’ to whether students should be assessed on professionalism: *‘It sort of feels like a sort of yes question to me. I’m not quite sure why somebody would say no’* [P4]. This led to discussion around the difficulty of defining professionalism and associated problems with assessment:

*…how do you teach professionalism? Is it something that's innate that you, you know, as a person in society, you meet this when you go to a restaurant or the doctors, or a dentist's office, you know something like that, or is it something that you have to be shown as we are all?* [P1]

A further issue was the placement of the professionalism assessment, with consensus that this should start as early as possible, e.g. during admissions or first year. 

## Discussion

The study successfully met its goal of understanding public views on professionalism assessment, methods, priorities, and sanctions — crucial insights as they represent current and future service users. SP participants, with more experience reflecting on student professionalism than the average layperson, provided contextually relevant insights. The blended method — having patient representatives reflect on public survey data — appears to be a novel approach.

A 1998 systematic review of patient priorities in General Practice ^49^ found that ‘humaneness’ and ‘competence/accuracy’ were most valued. Interestingly, 25 years later, survey respondents still prioritize ‘benevolence’ — akin to ‘humanity’ — and ‘competence’ as the top qualities for healthcare professionals ( Table 1 ^46^).

The survey responses most frequently highlighted human interaction as key elements to assess, with respect, good relationships, and honesty being the top priorities ( Table 2 ^46^). In the focus groups, respect, patient relationships, and trustworthiness were also emphasized. The challenge for students is well captured by the question of how to become an ‘empathic and approachable human being’.

The limitations of summative exams in assessing attitudes, such as empathy, are well known. In Clinical Communication, the challenge of evaluating empathy during OSCEs is common. A student might say, ‘I’m sorry to hear that’, to score well, but how can we know if it is genuine? The best judge may be the SP, trained to assess this. However, even if the student appears empathic in that moment, it’s just one moment in time. We cannot be sure they consistently empathise with patients or gauge their overall professionalism from a brief observation. Accurate assessment requires observing patterns over time, which is increasingly difficult with larger cohorts and limited placement opportunities in healthcare settings.

The concept of Professional Identity Formation (as explored in the introduction) is perhaps then more useful in that it frames professionalism as a process. A familiar tutor can assess elements of professionalism as they emerge in the individual, whilst acknowledging that the context is ever changing, and a career encompasses lifelong learning; there is no such thing as the ‘finished article’. Certainly, our results suggest public benevolence towards healthcare students who do not meet expected levels of professional behaviour. More than 54% saw this as an opportunity for feedback and reflection rather than for more punitive sanctions.

The extended group discussions about appearance and dress perhaps can be attributed to the amount of time spent by SPs in keenly observing the detail of students’ professionalism, reflecting their educational focus. Their expertise could perhaps be used more widely in advising educational programmes and governing bodies, and in formative and summative assessments. It may be that some emphasis has been picked up from examiners — anecdotally prone to focus on dress code — which reinforces the need for authentic public, and semi-institutionalised patients, to contribute to methodological development.

It is encouraging that 97% of respondents supported the assessment of professionalism. Notably, 40% favoured informal methods (verbal/written feedback or progress meetings with staff), while 31% preferred a combination of informal and formal assessments, reflecting SPs’ preference for a hybrid approach. Healthcare students consistently request more formative feedback opportunities, as seen in NSS data (restricted by our ethics approval). This supports the consensus mentioned in the introduction that a longitudinal approach is ideal ^5, 6, 14, 24, 25, 50^.

This study questions whether healthcare training should prioritize assessing professional identity formation over knowledge acquisition, suggesting the need for a balance. Despite various student activities (e.g., portfolios, reflective reports and clinical assessments), professional development is often assumed adequate unless issues like OSCE failure or fitness-to-practice investigations arise.

The study’s limitations include a small sample size, opportunistic research methods, and self-selecting focus groups. Consequently, conclusions are preliminary, potentially guiding further research and incorporating patient perspectives in assessments. We class this as a pilot and are seeking partners for comparisons and to expand the data.

## Conclusion and recommendations

Public support for assessing professionalism was strong, but the aspects valued by the public differed from those currently emphasized by assessors. SPs involved in assessment called for greater focus on professional development, integrating professionalism into teaching, and giving it more weight in exams. These results can guide educators in revising curricula and assessments across medical and healthcare courses. The findings are applicable across programmes and could inform larger-scale projects, including international comparisons, beyond the West Midlands, UK.

## Ethical and security consideration, data protection issue

Human data from focus group discussions cannot be made available for this article. Ethical approval limits sharing of raw data transcripts and the focus group participation information sheet states that the information recorded is confidential and no-one except the research team listed above will have access to the recordings, although transcripts (anonymized) will be stored in a secure, university server.

The data that support this study cannot be publicly shared due to ethical or privacy reasons and may be shared upon reasonable request to the corresponding author if appropriate. Requests can be submitted to: j.d.ward@bham.ac.uk. All requests will be reviewed by the research team to ensure ethical, or privacy considerations are not breached in accordance with the approvals provided by the University of Birmingham Ethics Committee.

## Data Availability

Figshare: Dataset for UoB Public and SP voice in the assessment of professionalism survey.csv. https://doi.org/10.6084/m9.figshare.29390663.v1 (Ward *et al.*, 2025b) ^45^. The project contains the following underlying data: Dataset for UoB Public and SP voice in the assessment of professionalism survey.csv Figshare: Public and SP voice in the assessment of professionalism quantitative data tables.docx. Quantitative survey tables. https://doi.org/10.6084/m9.figshare.29390819 (Ward *et al.*, 2025c) ^46^. The project contains the following underlying data: Public and SP voice in the assessment of professionalism quantitative data tables.docx Underlying data are available under the terms of the Creative Commons Attribution 4.0 International license (CC-BY 4.0). Figshare: Methodological items: UoB Public and SP voice in the assessment of professionalism: participant information / consent form, focus group discussion flow. https://doi.org/10.6084/m9.figshare.29390888 (Ward *et al.*, 2025d) ^48^. This project contains the following extended data: - 2023 professionalism research - informed consent for focus groups July 2023.docx - Public and SP voice in the assessment of professionalism_Discussion Flow.docx Figshare: UoB Public and SP voice in the assessment of professionalism survey. Survey questionnaire. https://doi.org/10.6084/m9.figshare.29390981 (Ward *et al.*, 2025a) ^44^. This project contains the following extended data: - UoB Public and SP voice in the assessment of professionalism FINAL survey - Google Forms.pdf Extended data are available under the terms of the Creative Commons Attribution 4.0 International license (CC-BY 4.0).
