# Supplementary material for: Public and Simulated Patient voice in the assessment of professionalism on healthcare degree programmes
Source: MedEdPublish (2016). 2025 Oct 13;15:206. [Version 1] doi: 10.12688/mep.21119.1 (PMC13662377; doi:10.12688/mep.21119.1)
Supplement: Peer review history

# Reviewer report: Reviewer response for version 1

**DOI:** 10.21956/mep.22623.r45509

**Published:** 2026-10-13

**Authors:** Magnus Hultin

**Affiliations:** 1 Umeå universitet, Umeå, Sweden

This article adds a public perspective to the discussion on how to assess professionalism in medical education. It is well written, problematised, and cites relevant literature.

 I have just a single question on the methods and some comments that might improve the manuscript's readability.

1. How was the data obtained from the oral survey analysed?
2. Table 3, the frequency table might be simplified as the number of respondents is 100. % and n are the same numbers.
3. Suggest rephrasing the argumentative “This study successfully met its goal of understanding” and “appears to be a novel approach” in the first paragraph of the discussion. The decision if it met its goal is up to the readers and also to decide if the approach appears to be novel.
4. Suggest rephrasing in the introduction “We hope that the findings” as it is speculative.

Have any limitations of the research been acknowledged?

Yes

Is the work clearly and accurately presented and does it cite the current literature?

Yes

If applicable, is the statistical analysis and its interpretation appropriate?

Yes

Are all the source data underlying the results available to ensure full reproducibility?

Yes

Is the study design appropriate and is the work technically sound?

Yes

Are the conclusions drawn adequately supported by the results?

Yes

Are sufficient details of methods and analysis provided to allow replication by others?

Partly

Reviewer Expertise:

Leadership and teamwork in acute care teams. Clinical trials.

I confirm that I have read this submission and believe that I have an appropriate level of expertise to confirm that it is of an acceptable scientific standard, however I have significant reservations, as outlined above.

# Reviewer report: Reviewer response for version 1

**DOI:** 10.21956/mep.22623.r44454

**Published:** 2025-12-24

**Authors:** Mohd Faizan Siddiqui

**Affiliations:** 1 Osh State University, Osh, Osh Region, Kyrgyzstan

The topic is timely and examines how public and simulated patient perceptions affect healthcare education professionalism assessment. The mixed-method approach of street surveys and focus-group talks contributes to Professional Identity Formation, assessment authenticity, and patient voice in competency evaluation debates. Although, its well-structured, well-written study has the potential for influencing medical education curriculum and research, but I had few concerns as well. Please find my comments below:

 1. The convenience sampling from a single public location introduces representativeness concerns. A more detailed acknowledgement of demographic limitations, potential selection bias, and how these factors may impact generalizability would improve transparency.

 2. Beyond sample size, please address constraints such as self-report bias, SP affiliation with academic settings, limited demographic profiling, and short survey engagement time. This will enhance study integrity.

 3. The discussion would benefit from a stronger synthesis, contrasting results with prior professionalism assessment frameworks, PIF theory, and student evaluation models and highlighting the novelty of the study.

 4. The narrative part of the introduction could be strengthened by presenting a concise operational definition at the start before expanding into the recent literature, thereby improving readability and conceptual framing.

 5. Kindly provide a bit more detail on survey design validity. It would be valuable to explain whether survey questions were piloted or validated and how ranking tasks were communicated to non-specialist participants to ensure comprehension and reliability.

 6. The results section may benefit from visual summarization, as currently the manuscript is verbose. Graphs, thematic concept maps, or comparative visuals could enhance reader understanding, especially where ranking data and focus-group themes intersect.

 7. The conclusion should adopt more cautious generalizations, given the localized and exploratory nature of the study.

 8. Check the manuscript for typos and grammatical errors.

 Good Luck

Have any limitations of the research been acknowledged?

Partly

Is the work clearly and accurately presented and does it cite the current literature?

Yes

If applicable, is the statistical analysis and its interpretation appropriate?

Not applicable

Are all the source data underlying the results available to ensure full reproducibility?

Yes

Is the study design appropriate and is the work technically sound?

Yes

Are the conclusions drawn adequately supported by the results?

Partly

Are sufficient details of methods and analysis provided to allow replication by others?

Yes

Reviewer Expertise:

1. Healthcare & Medicine 2. Medical Education 3. Public Health

I confirm that I have read this submission and believe that I have an appropriate level of expertise to confirm that it is of an acceptable scientific standard, however I have significant reservations, as outlined above.

# Reviewer report: Reviewer response for version 1

**DOI:** 10.21956/mep.22623.r43927

**Published:** 2025-11-08

**Authors:** Irma Manjavidze, Associate Professor. Dali Chitaishvili M.D., PhD.

**Affiliations:** 1 Tbilisi State Medical University, Tbilisi, Georgia; 2 Associate Professor at the Department of Clinical Skills, Tbilisi State Medical University, Tbilisi, Georgia

**General Comments**

**A well-designed study exploring public views on healthcare student professionalism through surveys and SP focus groups. It effectively links theory with practice, presents clear methods and results, and offers a thoughtful discussion on identity, assessment, and curricular reform. Minor notes follow.**

**Abstract**

- **SPs** should be spelled out as **Simulated Patients** at first mention for clarity.
- “Given that participants were recruited in a public right-of-way near a university and hospital, the use of a convenience sample is clear. However, it would still be helpful for readers if the authors briefly acknowledged this limitation and commented on any efforts (or lack thereof) to capture demographic diversity in the sample
- Keywords are inconsistently capitalized — apply consistent lowercase or title case (e.g., all lowercase is preferred by many journals).

**Introduction**

- Consider explicitly stating early on why incorporating public perspectives is valuable—e.g., to align educational goals with societal expectations.

**Methods**

- Sampling limitations need clearer acknowledgment upfront. For example, the convenience sample from a single high-traffic area may limit representativeness. Consider adding a sentence noting this in the methods section.
- The question asking respondents to rank nine types of professionalism assessment methods may be complex for a convenience sample of the general public, many of whom may not be familiar with these specific assessment techniques. Given this complexity, I wonder if the allotted 5–10 minutes per respondent was sufficient for thoughtful consideration. Clarifying whether terms were explained or simplified during data collection would help assess the reliability of these responses. If such measures were not taken, I suggest the authors discuss this as a potential interpretation limitation.
- Using professional SPs offers valuable insight due to their familiarity with professionalism assessment, but their close ties to the academic setting may introduce bias. The manuscript should acknowledge this and provide more detail on recruitment, diversity efforts, and how group dynamics or power imbalances were managed to enhance trustworthiness.

**Results**

- There is some ambiguity regarding the purpose of the focus groups: were participants reacting to the survey findings (as implied), or providing independent reflections? This needs to be explicitly clarified. The text states that Part 2 results were “informed by Part 1” and that focus group themes emerged independently — this contradiction may confuse readers.

The manuscript allows readers to infer which assessment methods participants were asked about by referencing examples throughout the text. However, it would be helpful to consolidate a complete list of these methods in one place (e.g., a table or summary), so readers can see the full scope more easily.

**Discussion**

- Some points (e.g., empathy in OSCEs or the influence of examiners on SP focus) are speculative but interesting. Frame these as areas for future research and add supporting references where possible.

**Conclusion**

 While the findings offer valuable insights that may be relevant across programs, they are based on a small, localized sample. Therefore, these results should be viewed as preliminary and indicative, with potential to inform larger-scale, multi-site, or international studies in the future.

**Additional Notes**

- The manuscript clearly outlines ethical and data protection considerations and provides access to survey datasets and related materials. While participant compensation (vouchers and travel reimbursement) is described, the manuscript does not include a funding statement. Although this may not be mandatory, I suggest the authors consider including a brief funding declaration or a statement clarifying whether external funding was received to enhance transparency and align with best reporting practices.

**In summary:**

 Well-designed and relevant pilot study with clear writing and solid methods, but needs clearer sampling details, focus group clarity, and more thorough discussion of limitations.

Have any limitations of the research been acknowledged?

Partly

Is the work clearly and accurately presented and does it cite the current literature?

Yes

If applicable, is the statistical analysis and its interpretation appropriate?

Yes

Are all the source data underlying the results available to ensure full reproducibility?

Partly

Is the study design appropriate and is the work technically sound?

Yes

Are the conclusions drawn adequately supported by the results?

Yes

Are sufficient details of methods and analysis provided to allow replication by others?

Partly

Reviewer Expertise:

medical education, simulation-based learning, OSCE

We confirm that we have read this submission and believe that we have an appropriate level of expertise to confirm that it is of an acceptable scientific standard.
